# Supplementary material for: Structural organization of p62 filaments and the cellular ultrastructure of calcium-rich p62-enwrapped lipid droplet cargo
Source: Nat Commun. 2025 Nov 28;16:10810. doi: 10.1038/s41467-025-66785-7 (PMC12669770; doi:10.1038/s41467-025-66785-7)
Supplement: Supplementary file 1 — Supplementary Information [file 41467_2025_66785_MOESM1_ESM.pdf]

# Structural organization of p62 filaments and the cellular ultrastructure of calcium-rich p62-enwrapped lipid droplet cargo

Sabrina Berkamp[#†], Lisa Jungbluth[#], Alexandros Katranidis[#], Siavash Mostafavi, Olivera Korculanin, Peng-Han Lu, Lotte Ickert, Maya M. Dierig, Lokesh Sharma, Lipi Thukral, Pitter F. Huesgen, Natalia L. Kononenko, Jörg Fitter, Rafal E. Dunin-Borkowski, Carsten Sachse[†]

[#]These authors contributed equally

[†] Correspondence: s.berkamp@fz-juelich.de (S.B.), c.sachse@fz-juelich.de (C.S.)

## Supplementary Information

**Supplementary Figure 1: p62 filament sample optimization, helical pitch of p62 filaments and resolution assessment of p62 cryo-EM structure.** ................................2

**Supplementary Figure 2: AlphaFold3 prediction of p62 assembly, domain localization, binding of LC3b and competition with ubiquitin.** ....................................3

**Supplementary Figure 3: Phase separation of p62 filaments induced by addition of GST-4xUbiquitin.** ....................................................................................................4

**Supplemental Figure 4: Confocal fluorescence microscopy analysis of lipid droplet size upon ATG5 knockdown in wildtype RPE1 cells.** ...................................................5

**Supplementary Figure 5: Different thicknesses of droplet coats.** ...............................6

**Supplementary Figure 6: Segmented tomogram showing a thin discontinuous high-contrast p62-positive coat surrounding the lipid droplet.** ............................................7

**Supplementary Figure 7: Energy-dispersive X-ray (EDX) spectroscopy combined with STEM imaging of p62-positive lipid droplets.** .......................................................8

**Supplementary Figure 8: Energy-dispersive X-ray spectroscopy (EDX) combined with STEM imaging of native lipid droplets** ..................................................................9

**Supplementary Figure 9: Energy-dispersive X-ray (EDX) spectroscopy combined with STEM imaging of a lysosome.** ..................................................................10

**Supplementary Figure 10: Effect of divalent cation salts on p62 filaments** ...............11

**Supplementary Figure 11: Angular distribution and helical symmetry (a) Angular distribution in final 3D helical reconstruction. (b) Helical symmetry error surface.** .12

**Supplementary Figure 12: Bubble-model of pH affecting double-helical filament organization and statistical significance analysis of the two p62 filament length datasets.** ............................................................................................................13

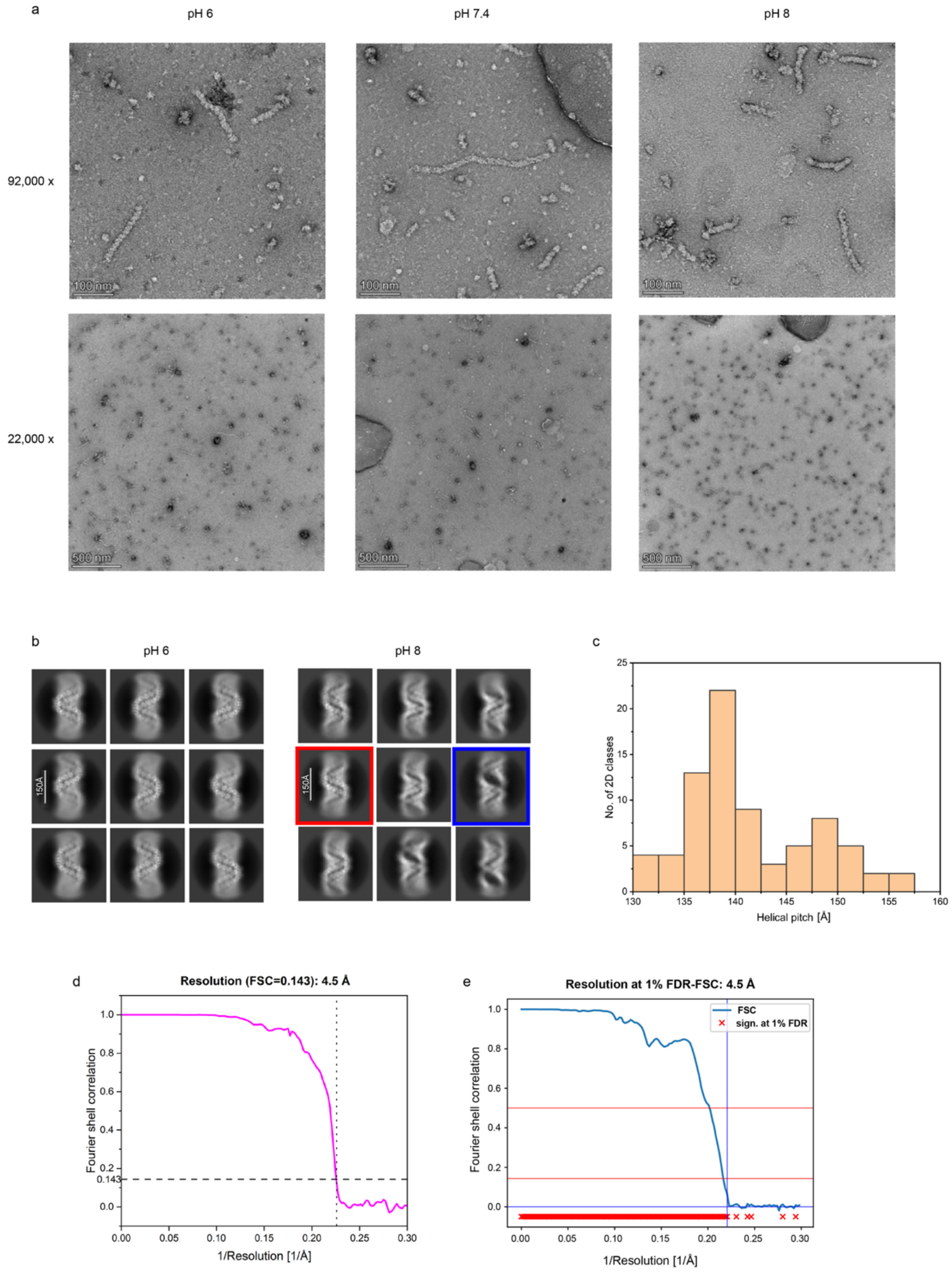


**Supplementary Figure 1: p62 filament sample optimization, helical pitch of p62 filaments and resolution assessment of p62 cryo-EM structure.**

(a) High and low magnification images of negatively stained p62 filaments formed at pH 6, 7.4 and 8. (b) 2D classification at pH 6 (left) and pH 8 (right). At pH 6, the classes show a regular helical architecture. At pH 8 almost 60% of the particles show an unwinding of the two strands (blue), whereas 40% of the particles still show a closed form of the filament (red). (c) Helical pitch distribution of p62 filaments ranges from 130 to 160 Å. (d) Fourier shell correlation (FSC) curve of the p62 filament density map indicates a resolution according to the cutoff FSC(0.143)=4.5 Å. (e) FDR-FSC curve indicates a resolution of 4.5 Å. Source data are provided as a Source Data file.

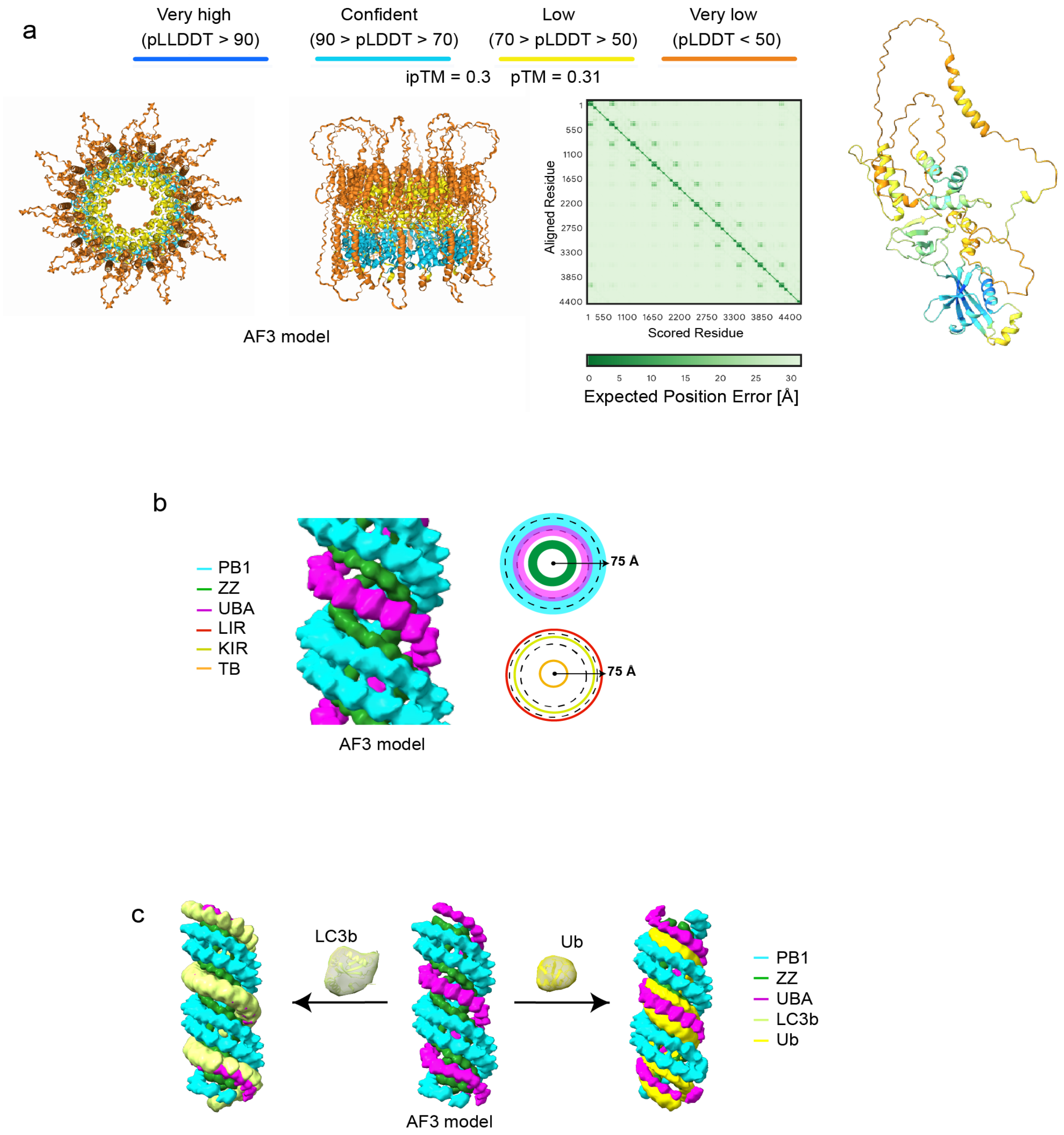


**Supplementary Figure 2: AlphaFold3 prediction of p62 assembly, domain localization, binding of LC3b and competition with ubiquitin.**
(a) Left. AlphaFold3 (AF3) model of the p62 decamer ring (top and side view), colored according to a per-atom confidence estimate. Predicted aligned error (PAE) plot reveals spatial proximity of the domains. Right. Single p62 molecule AF3 prediction excised from 10-mer colored with per-residue model confidence estimate. (b) p62 filament model of AF3 predictions showing PB1 (cyan), ZZ (green) and UBA (magenta) domains rendered at 15 Å resolution. Top views showing relative position of subunits and binding sites and their radial distance from the center of the filament. The two dotted lines mark the position of the PB1 domain that corresponds to the strongest observed density in the cryo-EM map. (c) p62 filament models of AF3 predictions rendered at 15 Å resolution with LC3b (left) or ubiquitin (right) bound. AlphaFold Server powered by AlphaFold 3 was used in June 2024.

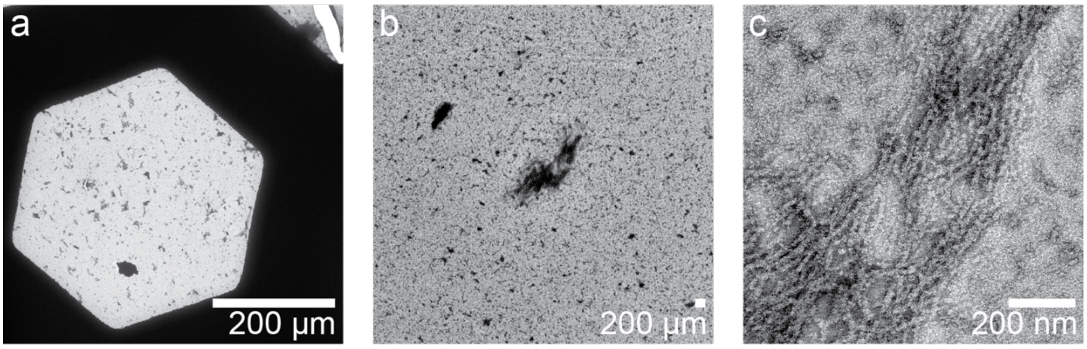


**Supplementary Figure 3: Phase separation of p62 filaments induced by addition of GST-4xUbiquitin.**

p62 filaments at 1 $\mu$M concentration were mixed with 15 $\mu$M GST-4xUbiquitin, incubated for four minutes and subsequently negatively stained. The resulting phase separation droplets were imaged at (a) 100x, (b) 8,500x and (c) 57,000x magnification revealing dense clusters of p62 filament bundles.

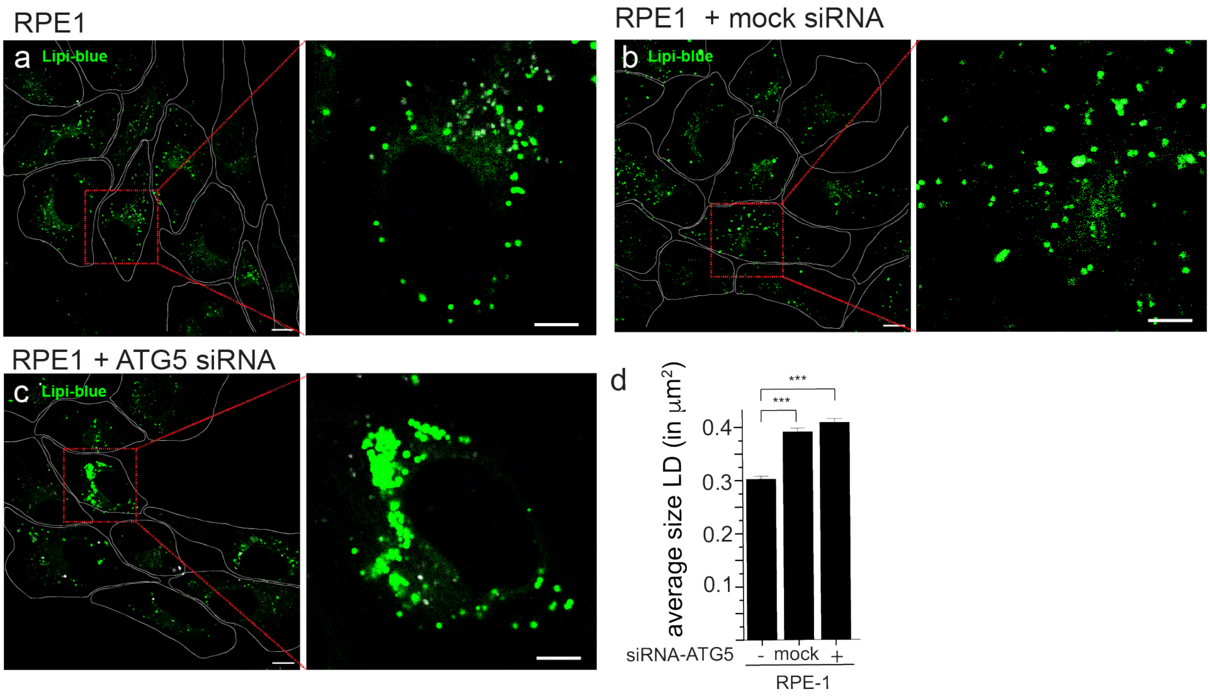


**Supplemental Figure 4: Confocal fluorescence microscopy analysis of lipid droplet size upon ATG5 knockdown in wildtype RPE1 cells.**

(a) Reference RPE1 cells stained with Lipi-Blue for 24 hours at conditions of (A) no transfection, (b) 72 hrs after a mock transfection with RNAiMax and (c) 72 hrs after a transfection with ATG5 siRNA. Quantifications of the average size of lipid droplets per cell. Statistical significance was determined with a two sample t-test: *****=p<0.001, data are presented as mean values as the length of the box and the standard error denoted with a whisker. For RPE1 cells 151, cells were analyzed, for mock transfected cells, 125 cells were analyzed and, for RPE1 cells with ATG5KD, 158 cells were analyzed, all over 3 independent biological replicates, with $p=3.726\text{-}10^{-43}$, and $p=8.608\text{-}10^{-56}$ between RPE1 and mock transfected cells and RPE1 and ATG5KD cells respectively. Scale bars all images 10 µm.

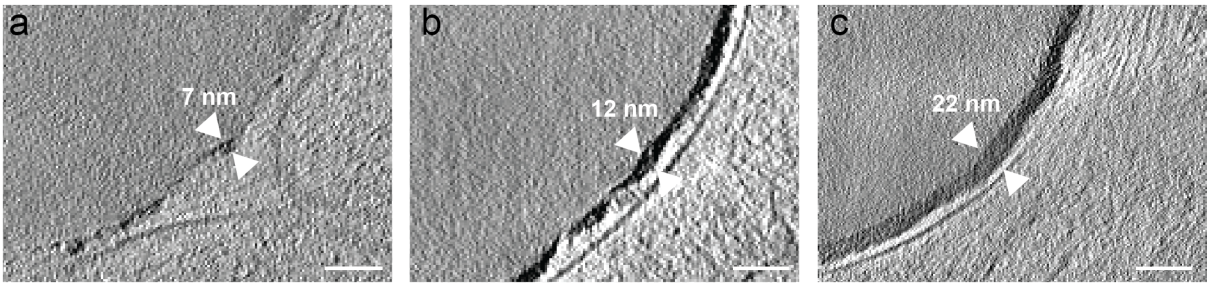


**Supplementary Figure 5: Different thicknesses of droplet coats.**
Lipid droplets are coated by a high-contrast layer of different thicknesses measuring (a) 7 nm, (b) 12 nm and (c) 22 nm. Scale bar 50 nm.

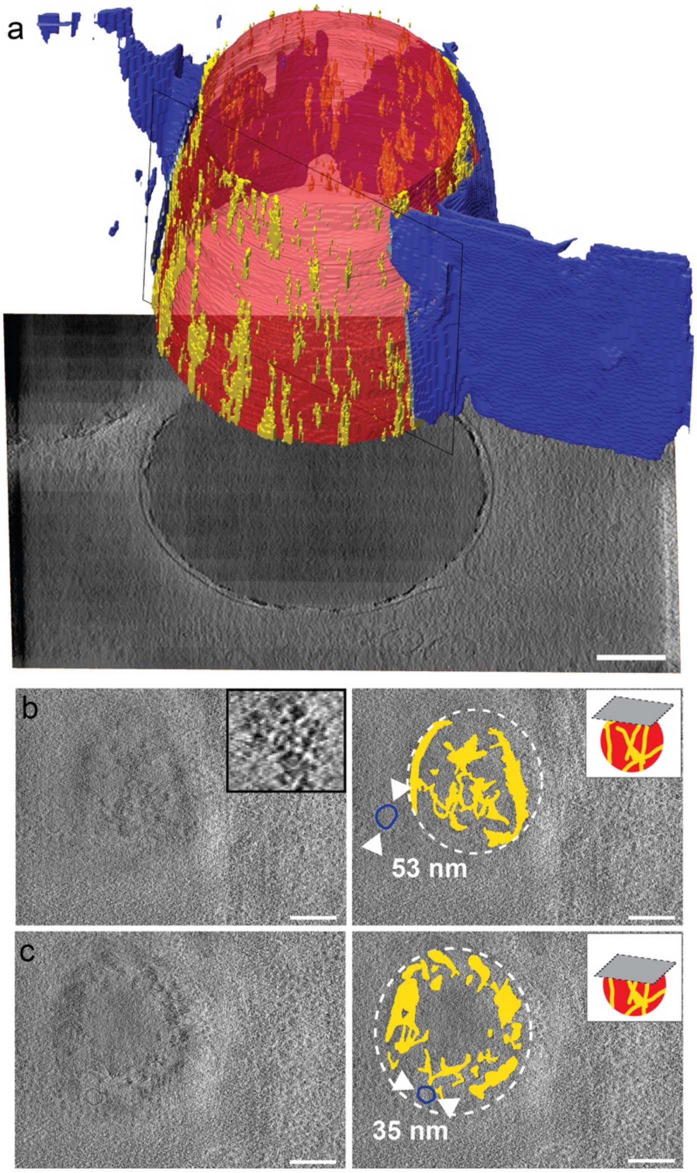


**Supplementary Figure 6: Segmented tomogram showing a thin discontinuous high-contrast p62-positive coat surrounding the lipid droplet.**
(a) Segmented tomogram rendered in three dimensions: lipid droplet (red), p62-positive coat (yellow) enwrapped by a single lipid bilayer stack (blue). The membrane was opened up using a clipping plane (black rectangle) for easier visualization. (b) Upper z-section through segmented tomogram reveals the thin discontinuous surface coating around the lipid droplet. Inset shows the granular nature of the surface coating. (c) Center z-section of the same lipid droplet. Small vesicular structures (blue) of 35 and 53 nm diameter are also visible in both z-slices. Scale bar 100 nm.

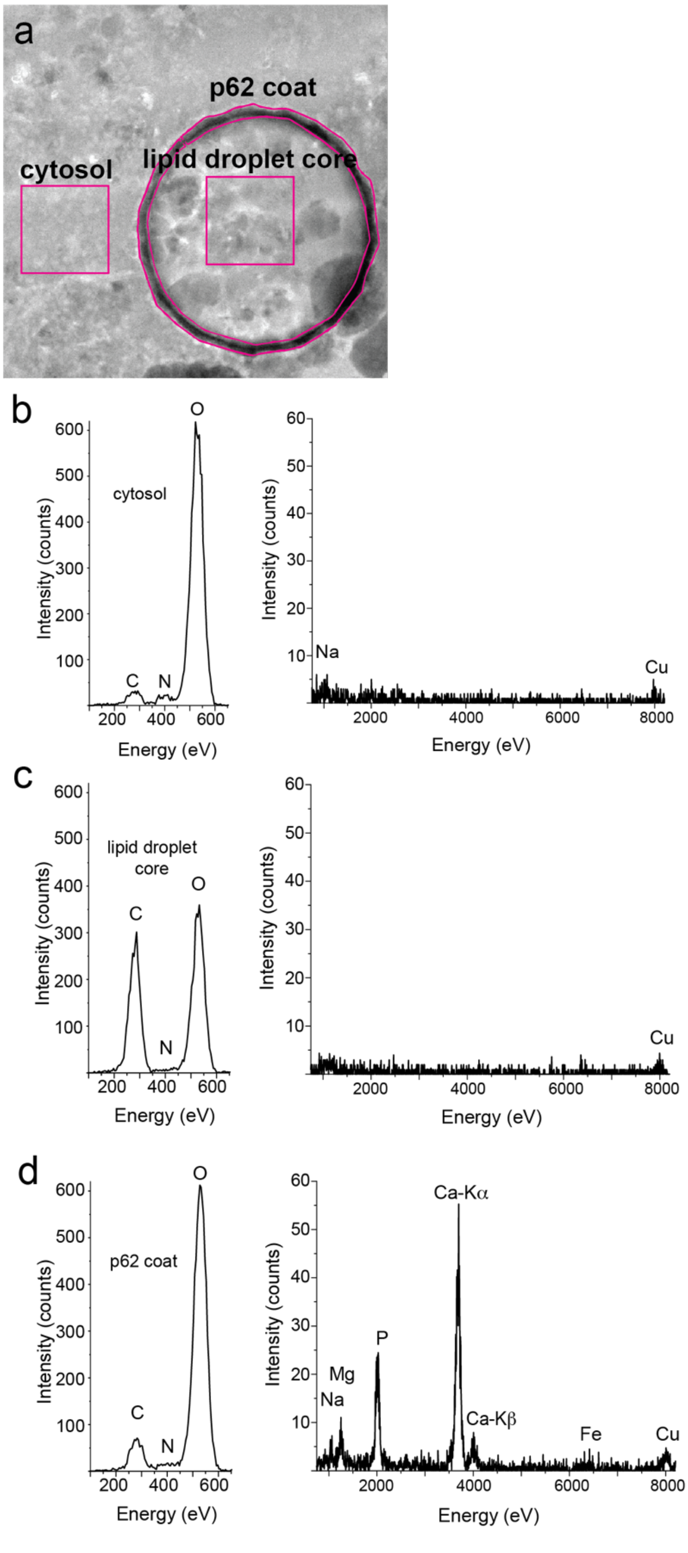


**Supplementary Figure 7: Energy-dispersive X-ray (EDX) spectroscopy combined with STEM imaging of p62-positive lipid droplets.**
(a) HAADF-STEM image of p62 positive lipid droplets. Insets indicate the regions used for summing of EDX signals. The same number of pixels were summed in all regions. X-ray spectra showing which elements are present in the different areas; in (b) cytosol, (c) lipid droplet core and (d) dense p62 positive coat, respectively. Peaks were marked with their respective elements. The weak copper (Cu) peak originates from the copper sample holder used.

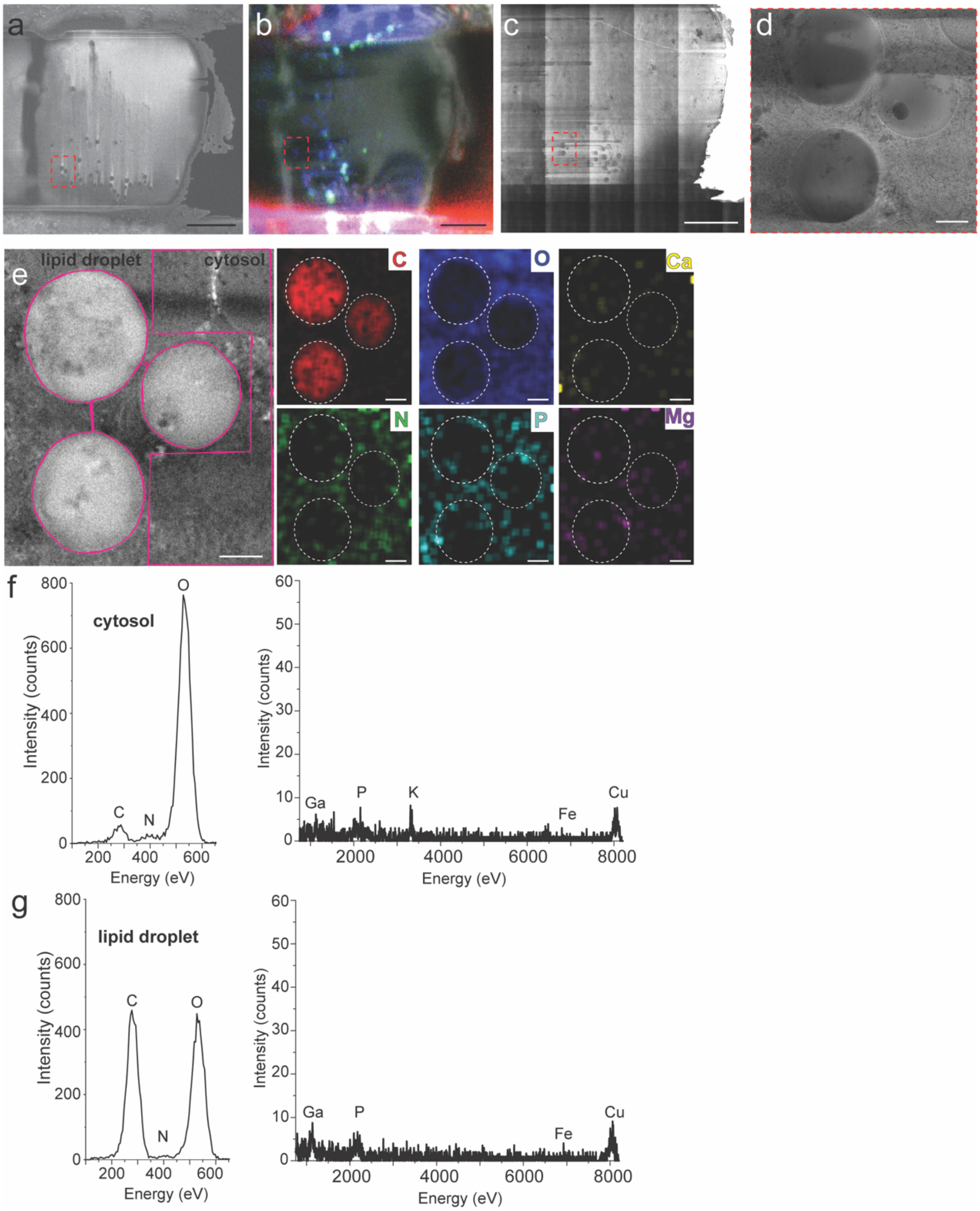


**Supplementary Figure 8: Energy-dispersive X-ray spectroscopy (EDX) combined with STEM imaging of native lipid droplets.**
(a) Scanning electron microscopy images of lamella with visible droplets in inset. Scale bar 5 $\mu$m. (b) SEM image superimposed with fluorescent image of Lipi-Blue dye. (c) Overview image of corresponding recorded transmission electron microscope (TEM). (d) Three spatially close lipid droplets in higher magnification TEM image. (e) HAADF-STEM image of three adjacent lipid droplets corresponding elemental images of carbon (C), oxygen (O), calcium (Ca), nitrogen (N), phosphorus (P) and magnesium (Mg). The image shows two insets of the cytosol and droplet region used for subsequent averaging. EDX spectra of (f) cytosol region and (g) lipid droplet regions. Scale bar 200 nm. Experiments were done in one session, on 3 independent cellular lamellae and yielded similar results.

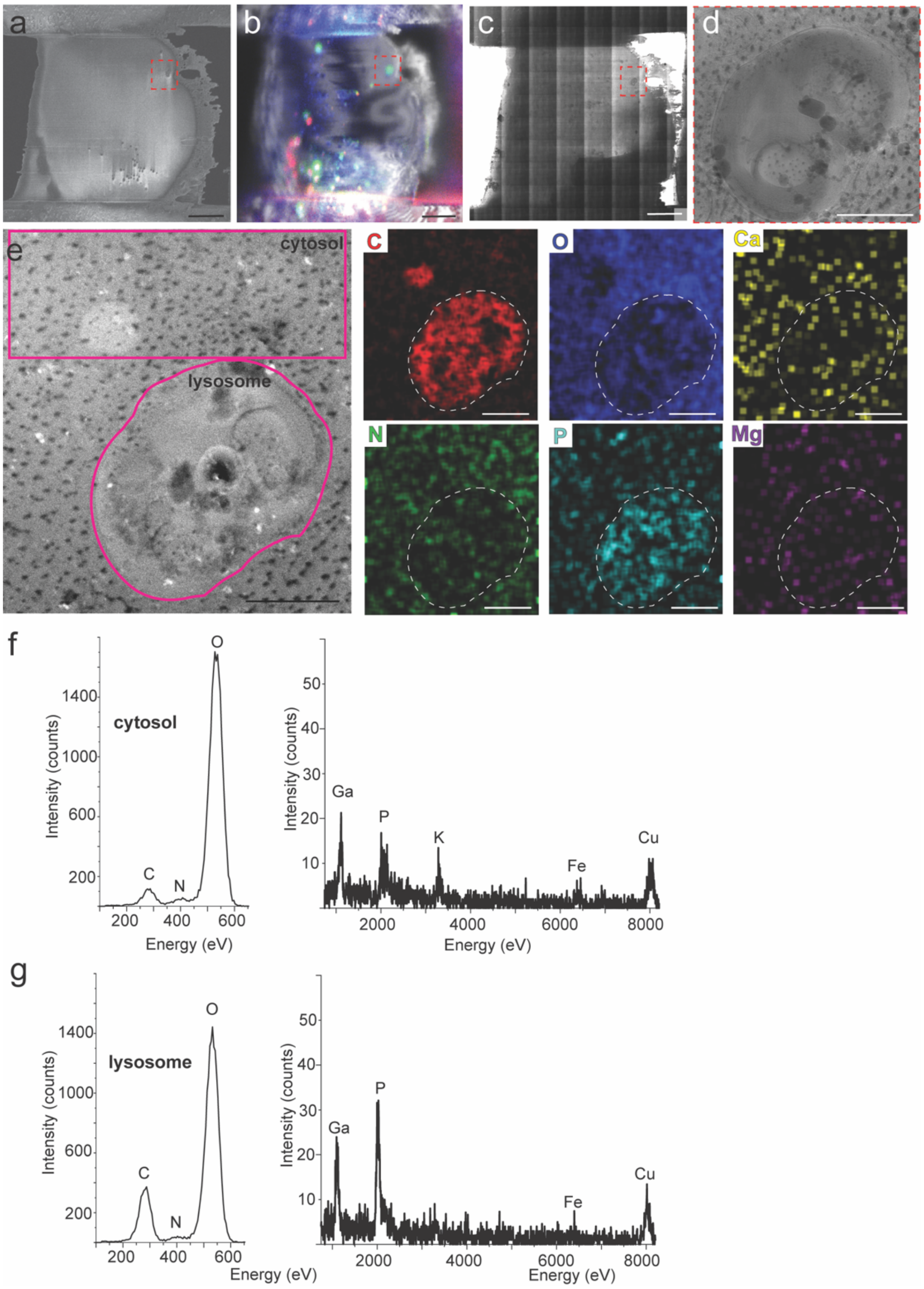


**Supplementary Figure 9: Energy-dispersive X-ray (EDX) spectroscopy combined with STEM imaging of a lysosome.**

(a) Scanning electron microscopy (SEM) images of lamella with visible droplets in inset. (b) SEM image superimposed with fluorescent image of green autofluorescence. (c) Overview image of corresponding recorded transmission electron microscope (TEM). (d) Large vesicular lysosome structure in higher magnification TEM image. (e) HAADF-STEM image of lysosome corresponding elemental images of carbon (C), oxygen (O), calcium (Ca), nitrogen (N), phosphorus (P) and magnesium (Mg). Scale bar 500 nm. The image shows two insets of the cytosol and droplet region used for subsequent averaging. EDX spectra of (f) cytosol region and (g) lysosome regions show significant gallium signal originating from the FIB preparation procedure. Experiments were done in one session, on 2 independent cellular lamellae and yielded similar results.

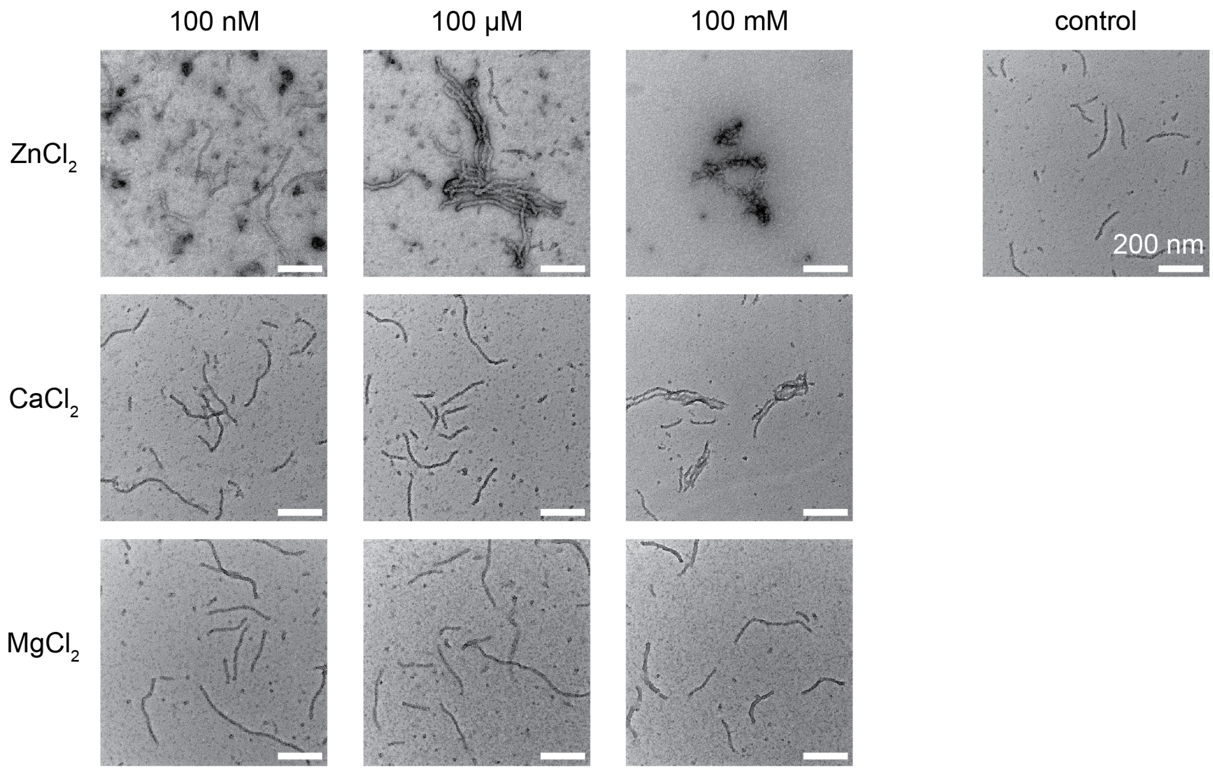


**Supplementary Figure 10: Effect of divalent cation salts on p62 filaments.**
p62 filaments at 1 $\mu$M concentration were mixed with 100 nM, 100 $\mu$M and 100 mM of $ZnCl_2$, $CaCl_2$ and $MgCl_2$, respectively. The samples were incubated for 30 minutes and subsequently negatively stained. As a control, p62 filaments were mixed with desalting buffer. For increasing concentrations of $ZnCl_2$ from 100 $\mu$M and for $CaCl_2$ from 100 mM bundling of p62 filaments was observed. For $MgCl_2$ no clear effect of bundling was observed.

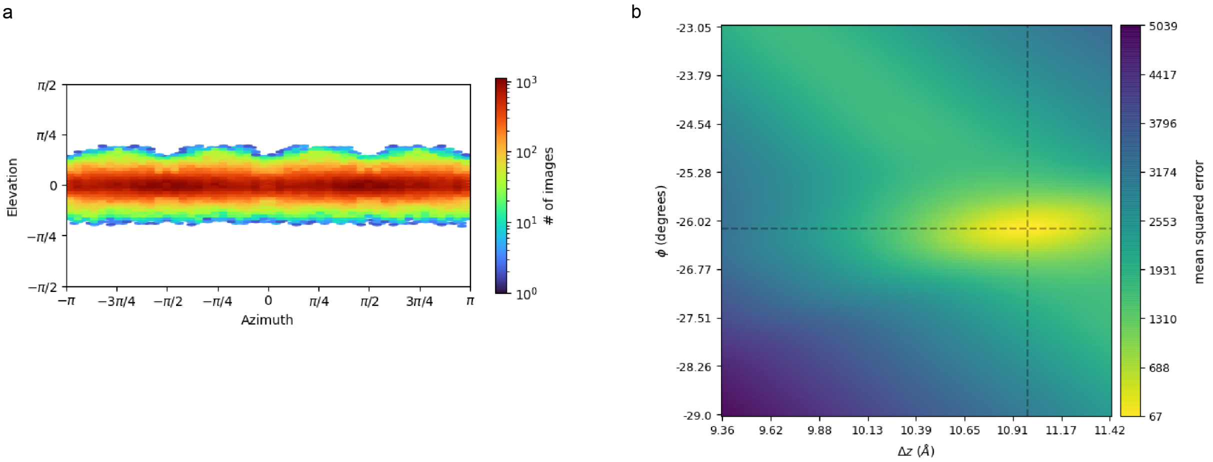


**Supplementary Figure 11: Angular distribution and helical symmetry** (a) Angular distribution in final 3D helical reconstruction. (b) Helical symmetry error surface.

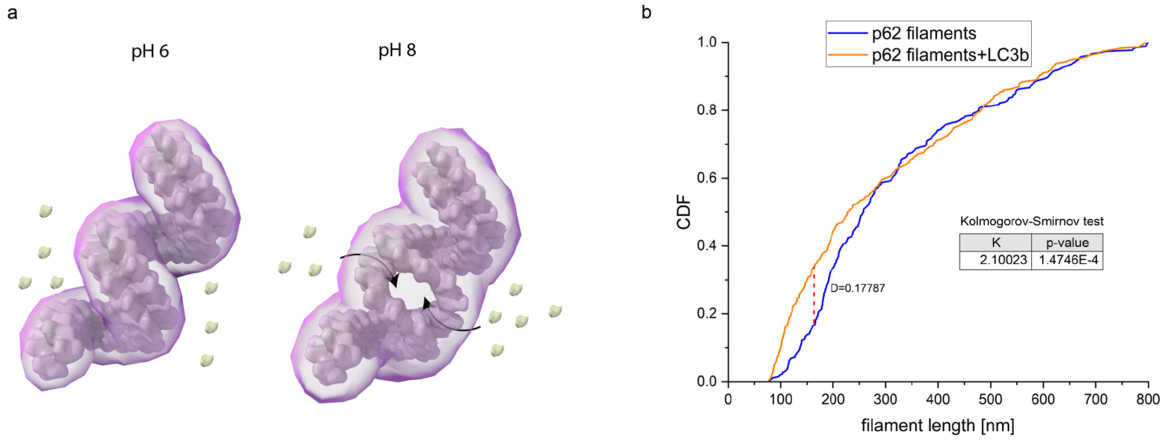


**Supplementary Figure 12: Bubble-model of pH affecting double-helical filament organization and statistical significance analysis of the two p62 filament length datasets.**
(a) At pH 6 we observed a closed double-helical filament whereas closer to physiological pH at pH 8 almost 60 % showed partially opened PB1 domain strands potentially resulting in improved access for p62 binding partners. (b) The cumulative density distribution (CDF) was calculated for p62 filaments without LC3b (blue line) and p62 filaments in the presence of LC3b (orange line). The dashed red line gives the maximum absolute difference between the two CDFs, D=0.17787. The Kolmogorov-Smirnov test statistic was calculated K= 2.10023 and the approximate p-value was derived p= 0.00014746. Since $p<0.05$ the observed shift of the distribution in the presence of LC3b towards shorter filament lengths is statistically significant.
