# Supplementary material for: Structural organization of p62 filaments and the cellular ultrastructure of calcium-rich p62-enwrapped lipid droplet cargo
Source: Nat Commun. 2025 Nov 28;16:10810. doi: 10.1038/s41467-025-66785-7 (PMC12669770; doi:10.1038/s41467-025-66785-7)
Supplement: Supplementary file 2 — Description of Additional Supplementary Files [file 41467_2025_66785_MOESM2_ESM.pdf]

Description of Additional supplementary files

Supplementary Movie 1: p62 filaments visualized by TIRF microscopy. p62 filaments labeled with Cy5 were visualized using the red channel of the TIRF microscope. They are rather mobile and diffusing above the surface.

Supplementary Movie 2: p62 filaments with LC3b visualized by TIRF microscopy. Upon addition of equimolar amounts of CFP-LC3b, labeled p62 filaments that are visible on the red channel become also visible on the blue channel when excitation laser changes from red (639 nm) to blue (488 nm).

Supplementary Movie 3: p62 filaments with GST-4xUb form µm-sized condensates. Upon addition of G ST-4xUb to the labeled p62 filaments, rapid and spontaneous formation of µm-sized condensates takes place displaying minimal mobility.

Supplementary Movie 4: p62 GST-4xUb condensates are dissolved by LC3b. Subsequent addition of CFP-LC3b to the formed condensates results in the appearance of smaller mobile diffusing fragments
