# Supplementary material for: Structural organization of p62 filaments and the cellular ultrastructure of calcium-rich p62-enwrapped lipid droplet cargo
Source: Nat Commun. 2025 Nov 28;16:10810. doi: 10.1038/s41467-025-66785-7 (PMC12669770; doi:10.1038/s41467-025-66785-7)
Supplement: Supplementary file 7 — Reporting Summary [file 41467_2025_66785_MOESM7_ESM.pdf]

nature portfolio

Corresponding author(s): Sabrina Berkamp, Carsten Sachse

Last updated by author(s): Oct 24, 2025

Reporting Summary

Nature Portfolio wishes to improve the reproducibility of the work that we publish. This form provides structure for consistency and transparency in reporting. For further information on Nature Portfolio policies, see our Editorial Policies and the Editorial Policy Checklist.

Statistics

For all statistical analyses, confirm that the following items are present in the figure legend, table legend, main text, or Methods section.

n/a	Confirmed	
☐	☒	The exact sample size (*n*) for each experimental group/condition, given as a discrete number and unit of measurement
☐	☒	A statement on whether measurements were taken from distinct samples or whether the same sample was measured repeatedly
☐	☒	The statistical test(s) used AND whether they are one- or two-sided<br>*Only common tests should be described solely by name; describe more complex techniques in the Methods section.*
☒	☐	A description of all covariates tested
☐	☒	A description of any assumptions or corrections, such as tests of normality and adjustment for multiple comparisons
☒	☐	A full description of the statistical parameters including central tendency (e.g. means) or other basic estimates (e.g. regression coefficient) AND variation (e.g. standard deviation) or associated estimates of uncertainty (e.g. confidence intervals)
☐	☒	For null hypothesis testing, the test statistic (e.g. *F*, *t*, *r*) with confidence intervals, effect sizes, degrees of freedom and *P* value noted<br>*Give P values as exact values whenever suitable.*
☒	☐	For Bayesian analysis, information on the choice of priors and Markov chain Monte Carlo settings
☒	☐	For hierarchical and complex designs, identification of the appropriate level for tests and full reporting of outcomes
☒	☐	Estimates of effect sizes (e.g. Cohen's *d*, Pearson's *r*), indicating how they were calculated

Our web collection on statistics for biologists contains articles on many of the points above.

Software and code

Policy information about availability of computer code

Data collection	EPU (v2.12.1.2782), Serial EM (v3.7.0 beta), Zeiss ZEN, TEM Imaging & Analysis Software (TIA), MAPS(v3.19)
Data analysis	CryoSPARC (v3.3.2), Chimera (v1.16), ChimeraX (v1.5.dev202206170050), Coot (v0.98), Phenix (v1.20.1-4487), ImageJ (Version 1.54p), OriginPro (v10.0.0.154), CellProfiler (v4.2.6), Amira3D (v2022.2), Velox(v3.14), WARP(1.0.9), AreTomo(v2), IsoNet(v0.2), CryoCARE(0.3.0), MemBrainseg(v9b), Relion(4.0.1), MSConvertGUI (v3.0.21229-9668f52), FragPipe(v22.0), RStudio(v4.4.0)

For manuscripts utilizing custom algorithms or software that are central to the research but not yet described in published literature, software must be made available to editors and reviewers. We strongly encourage code deposition in a community repository (e.g. GitHub). See the Nature Portfolio guidelines for submitting code & software for further information.

Data

Policy information about availability of data

All manuscripts must include a data availability statement. This statement should provide the following information, where applicable:

- Accession codes, unique identifiers, or web links for publicly available datasets
- A description of any restrictions on data availability
- For clinical datasets or third party data, please ensure that the statement adheres to our policy

The EMDB accession number for the p62 cryo-EM map is EMD-52134 and the corresponding PDB-ID 9HGE for the fitted PB1 coordinates. The mass spectrometry

proteomics data have been deposited to the ProteomeXchange Consortium via the PRIDE [PubMed ID: 39494541] partner repository with the dataset identifier PXD066163 (Reviewer access details: Project accession: PXD066163, Token: 4GL1LPGCCXKk).

Research involving human participants, their data, or biological material

Policy information about studies with human participants or human data. See also policy information about sex, gender (identity/presentation), and sexual orientation and race, ethnicity and racism.

Reporting on sex and gender	N/A
Reporting on race, ethnicity, or other socially relevant groupings	N/A
Population characteristics	N/A
Recruitment	N/A
Ethics oversight	N/A

Note that full information on the approval of the study protocol must also be provided in the manuscript.

Field-specific reporting

Please select the one below that is the best fit for your research. If you are not sure, read the appropriate sections before making your selection.

☒ Life sciences ☐ Behavioural & social sciences ☐ Ecological, evolutionary & environmental sciences

For a reference copy of the document with all sections, see nature.com/documents/nr-reporting-summary-flat.pdf

Life sciences study design

All studies must disclose on these points even when the disclosure is negative.

Sample size	A total of 4038 movies were collected for the cryo-EM structure determination of p62. For the CryoET several hundred tomograms were collected, and the ones without our feature of interest discarded, leaving ~25 tomograms. For the confocal microscopy triplicates were done in three different sessions, per condition and per replicate 10-20 images were taken, with 2-8 cells per frame. For the mass spectrometry analysis quadruplicates of each sample were performed. For the EDX experiments, spectra were recorded on four p62 enwrapped lipid droplets.
Data exclusions	Micrographs of poor particle coverage and ice quality were discarded. Tomograms of poor ice quality were discarded, as well as tomograms without our feature of interest. For the confocal microscopy only cells were selected that were fully in the frame of view and cells for which the cell boundary could be clearly seen, this generally excluded cells in larger clusters.
Replication	Due to the time-consuming nature of image acquisition and the limited access to this specialized microscope equipment, exact replicates were not performed for the cryo-electron microscopy, cryo-ET and EDX. Triplicates were done for the confocal microscopy on RPE1 cells. Quadruplicates were done for the mass spectrometry
Randomization	N/A
Blinding	N/A

Reporting for specific materials, systems and methods

We require information from authors about some types of materials, experimental systems and methods used in many studies. Here, indicate whether each material, system or method listed is relevant to your study. If you are not sure if a list item applies to your research, read the appropriate section before selecting a response.

Materials & experimental systems

n/a	Involved in the study
☐	☒ Antibodies
☐	☒ Eukaryotic cell lines
☒	☐ Palaeontology and archaeology
☐	☒ Animals and other organisms
☒	☐ Clinical data
☒	☐ Dual use research of concern
☒	☐ Plants

Methods

n/a	Involved in the study
☒	☐ ChIP-seq
☒	☐ Flow cytometry
☒	☐ MRI-based neuroimaging

Antibodies

Antibodies used	The following antibodies were used: polyclonal guinea pig anti-p62 (1:1000, Progen, cat no GP62-C), Goat anti-Guinea Pig Alexa Fluor568 (1:1000, Invitrogen, cat no A-11075), Donkey anti-Rabbit Alexa Fluor 647 (1:1000, Invitrogen, cat no A-31573), polyclonal rabbit anti-p62 (1:1000 MBL International, Cat No PM045), monoclonal rabbit anti-ATG5 (1:5000, Abcam, Cat No ab108327), monoclonal mouse anti-GAPDH (1:1000, Invitrogen, Cat No MA1-16757), polyclonal rabbit anti-LC3 (1:500, Abcam, Cat No ab48394), goat anti-mouse HRP conjugate (1:2000, ThermoFisher Cat No 32230), and goat anti-rabbit HRP conjugate (1:2000, ThermoFisher, Cat No 31460).
Validation	*Describe the validation of each primary antibody for the species and application, noting any validation statements on the manufacturer's website, relevant citations, antibody profiles in online databases, or data provided in the manuscript.*

Eukaryotic cell lines

Policy information about cell lines and Sex and Gender in Research

Cell line source(s)	human hTERT RPE-1
Authentication	purchased directly from ATCC
Mycoplasma contamination	mycoplasma testing done yearly by sending the supernatant to be analyzed for mycoplasma contamination (analysis performed by Eurofins)
Commonly misidentified lines (See ICLAC register)	*Name any commonly misidentified cell lines used in the study and provide a rationale for their use.*

Animals and other research organisms

Policy information about studies involving animals; ARRIVE guidelines recommended for reporting animal research, and Sex and Gender in Research

Laboratory animals	Mus Musculus, C57Bl/6, both sexes, postnatal days P1-5,12-13 weeks old. Mice were maintained in a pathogen-free environment in ventilated polycarbonate cages. Animals were housed in groups of five animals per cage with constant temperature and humidity at 12h/12h light/dark cycles. Food and water were provided ad libitum.
Wild animals	This study did not involve wild animals
Reporting on sex	Initial observations indicated lack of sex-specific effect. In accordance with the SAGER guidelines, we have ensured that both male and female mice were included in all experimental groups.
Field-collected samples	N/A
Ethics oversight	All animal experiments were reviewed and approved by the ethics committee of the "Landesamtes für Natur, Umwelt- und Verbraucherschutz des Landes Nordrhein-Westfalen", Cologne (AZ 81-02.04.2020.A418, AZ 81-02-.04.2021.A067, AZ 81-02.04.2022.A116, AZ 81-02.04.2021.A067, AZ 81-02.04.2023.VG076, AZ 81-02.04.4.20.021).

Note that full information on the approval of the study protocol must also be provided in the manuscript.

Plants

Seed stocks	N/A
Novel plant genotypes	N/A
Authentication	N/A
