# Supplementary material for: Structural organization of p62 filaments and the cellular ultrastructure of calcium-rich p62-enwrapped lipid droplet cargo
Source: Nat Commun. 2025 Nov 28;16:10810. doi: 10.1038/s41467-025-66785-7 (PMC12669770; doi:10.1038/s41467-025-66785-7)

**Structural organization of p62 filaments and the cellular ultrastructure of calcium-rich p62-enwrapped lipid droplet cargo**

Sabrina Berkamp, Lisa Jungbluth, Alexandros Katranidis, Siavash Mostafavi, Olivera Korculanin, Peng-Han Lu, Lotte Ickert, Maya M. Dierig, Lokesh Sharma, Lipi Thukral*,* Pitter F. Huesgen, Natalia L. Kononenko*,* Jörg Fitter, Rafal E. Dunin-Borkowski, Carsten Sachse

**Uncropped blots:**

Figure 4A:

Anti-p62 blot


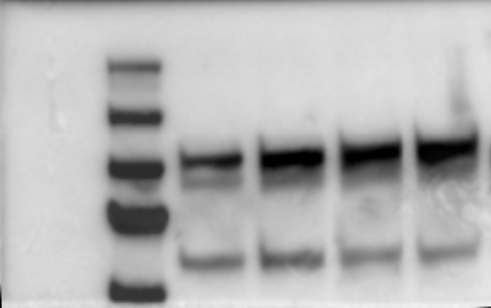


250

130

100

70

55

Anti-ATG5 blot:


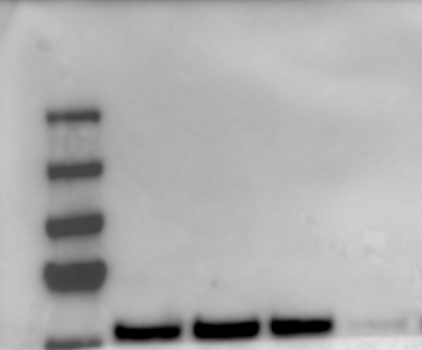


250

130

100

70

55

Anti GAPDH blot:


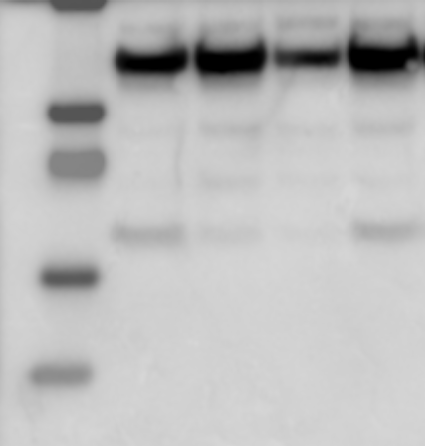


35

55

25

15

10

Uncropped blots:

Figure 7C:

Anti-p62 blot:


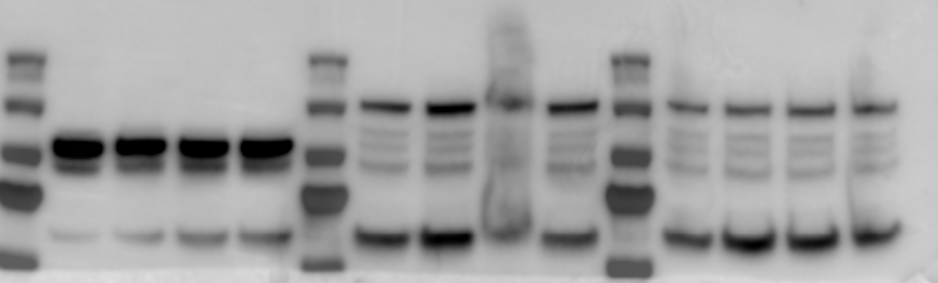


250

130

100

70

55

Anti-ATG5 blot:

250

130

100

70

55


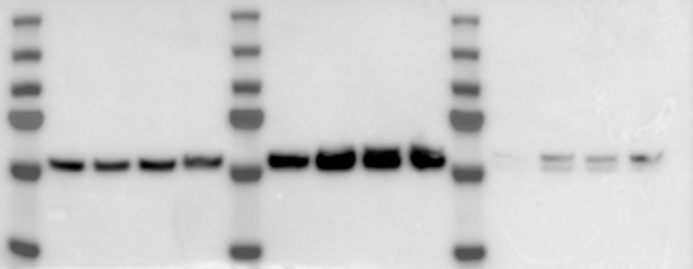


35

Anti-GAPDH blot:


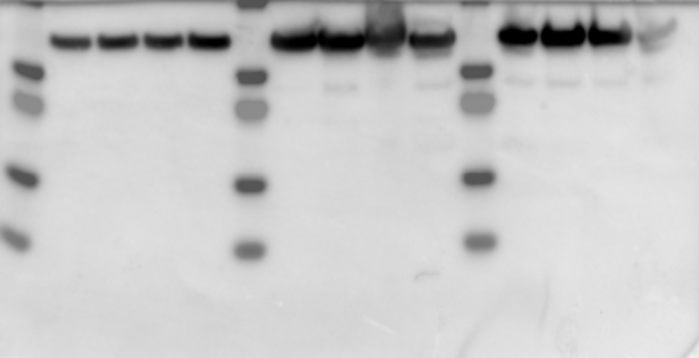


35

55

25

15

10

Anti-LC3 blot:

25

15

10


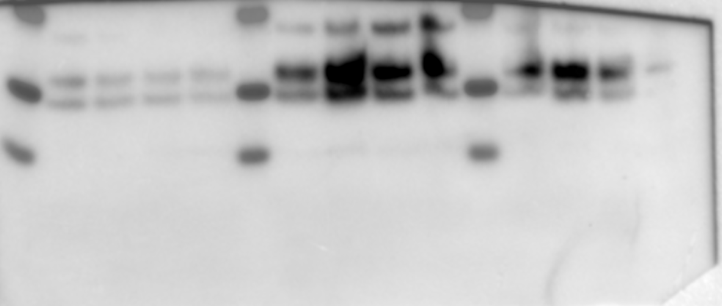

Supplement: Supplementary file 10 — Source Data 2 [file 41467_2025_66785_MOESM10_ESM.docx]
